# Outcome of HARMONIC Shears for lower limb free flap reconstruction in trauma: A retrospective cohort study

**DOI:** 10.1016/j.jpra.2025.12.024

**Published:** 2026-01-07

**Authors:** Mai Nishijo, Rawan Jaibaji, Calver Pang, Charles Yuen Yung Loh

**Affiliations:** a Department of Plastic and Plastic Reconstructive Surgery, Addenbrookes Hospital, Cambridge University Hospitals, Hills Road, Cambridge, UK; b Department of Surgical Biotechology, Division of Surgery and Interventional Science, Faculty of Medical Sciences, University Collegel London, Rowland Hill Street, London, UK

**Keywords:** Free tissue flaps, Reconstructive surgical procedures, Leg injuries, Ultrasonic surgical procedures, Cost-benefit analysis

## Abstract

**Background:**

The HARMONIC Shears is an ultrasonic surgical device enabling precise tissue dissection with simultaneous hemostasis. Its application in lower limb free flap reconstruction following trauma is underexplored.

**Methods:**

A retrospective cohort study compared nine patients undergoing lower limb free flap reconstruction with HARMONIC FOCUS™+ Shears to nine patients using conventional dissection (diathermy and LIGACLIP clips). Patients were matched by flap type and concomitant orthopedic procedures. Primary outcome was total operative time. Secondary outcomes included flap survival, postoperative complications, length of hospital stay, and a comprehensive cost analysis encompassing theater time, consumables, and inpatient stay.

**Results:**

HARMONIC Shears significantly reduced operative time in cases without concomitant orthopedic procedures (262.8 ± 46.3 vs 364.2 ± 53.9 min; *p* = 0.012). Across all cases, mean operative time was shorter in the HARMONIC Shears group (322.0 ± 81.1 vs 380.4 ± 53.5 min; *p* = 0.09). Flap survival was higher with HARMONIC Shears, with higher reoperation rates in the conventional group reflecting flap failures; however, this difference was not statistically significant. Specific postoperative complications rates, including infection, wound dehiscence, and donor site morbidity, were slightly higher in the HARMONIC Shears group, but all differences were not statistically significant. Mean hospital stay was 8.5 days shorter in the HARMONIC Shears group (*p* = 0.31). Cost analysis revealed theater and inpatient savings of up to £8699 per case with the HARMONIC Shears after accounting for device costs.

**Conclusions:**

HARMONIC Shears may enhance operative efficiency and reduce healthcare costs in lower limb free flap reconstruction without compromising clinical outcomes. However, findings should be interpreted cautiously given the small sample size and limited statistical power. Larger prospective studies are warranted to confirm these findings.

## Introduction

The HARMONIC Shears is an advanced surgical instrument that utilizes high-frequency ultrasonic vibrations (approximately 55,000 Hz) to simultaneously cut and coagulate tissue. The mechanical energy generated by the vibrating blade induces cellular disruption for precise tissue dissection, while the resulting frictional heat denatures proteins, forming a coagulum to achieve hemostasis.^1^ Operating at lower temperatures than conventional electrocautery, it minimizes lateral thermal spread and reduces collateral tissue damage.^2^^,^^3^ Although widely adopted in several surgical disciplines, including general, pediatric, gynecological, urological, and thoracic surgery, its utilization within plastic and reconstructive surgery is scarce.

Among the various models available, the HARMONIC FOCUS™+ Shears (Ethicon, Johnson & Johnson MedTech, Somerville, NJ, USA) is specifically designed for open procedures requiring fine dissection and precise control in confined anatomical spaces. Its slim profile and curved, fine blade tips allow for meticulous dissection near critical neurovascular structures, while enabling vessel sealing of up to 5 mm in diameter.^4^ These properties make it particularly advantageous for flap elevation and perforator dissection in microsurgical reconstruction.

Conventional methods of securing branch ligation during flap harvest include the use of LIGACLIPS™ (Ethicon, Johnson & Johnson MedTech, Somerville, NJ, USA) either deployed manually or using a LIGACLIP applicator. This results in multiple change of instruments and often double clipping of larger branches for fear of blood leaking. Other smaller vessel branches are often secured with traditional bipolar forceps but can result in rebleeds as well and are only suitable for small branches.

A recent meta-analysis by Kim et al.^5^ assessed the efficacy of ultrasonic shears vs conventional electrocautery in free flap surgery, demonstrating that the HARMONIC Shears significantly decreased flap harvest time and postoperative drain output, thereby suggesting both intraoperative and postoperative benefits. However, most studies to date have focused on applications in head and neck reconstruction,^6^, ^7^, ^8^, ^9^ covering flap types such as the radial forearm free flap (RFFF),^6^^,^^7^ fibular free flap^6^, ^7^, ^8^^,^^10^ and anterolateral thigh (ALT) flap,^8^^,^^11^ primarily for oncologic indications. The device has also been evaluated in breast reconstruction, particularly for the deep inferior epigastric perforator (DIEP) flap, where reductions in operative and flap elevation time have been observed.^12^

Despite encouraging results in these domains, the use of the HARMONIC Shears in lower limb trauma reconstruction remains underexplored. To date, there is no published data assessing its role in raising flaps such as the ALT flap for lower limb soft tissue coverage following trauma. This represents a significant gap in the literature, especially considering the potential for operative time reduction, cost savings, and improved hemostasis in complex trauma settings, where lengthy surgeries can be associated with higher complication rates and increased healthcare resource utilization.

This study aimed to evaluate the impact of the HARMONIC Shears on operative efficiency, clinical outcomes, and cost-effectiveness in lower limb free flap reconstruction following trauma. The primary objective was to compare operative duration between procedures performed using the HARMONIC Shears and conventional dissection techniques. Secondary objectives included the assessment of surgical outcomes, such as flap survival, postoperative complications, and reoperation rates, as well as an estimation of cost differences, incorporating theater time, consumable use and length of inpatient stay.

## Method

### Study design

A retrospective review was conducted using the departmental database at the Department of Plastic and Reconstructive Surgery, Cambridge University Hospitals NHS Foundation Trust. Patients who underwent free flap reconstruction for lower limb trauma between January 1 2024 and June 30 2025 were retrospectively identified from a prospectively maintained institutional database.

Patients were categorized into two groups according to the surgical technique used for flap harvest and pedicle dissection. The first group consisted of patients in whom the HARMONIC FOCUS™+ Shears (Ethicon, Johnson & Johnson MedTech, Somerville, NJ, USA) was used. A comparator cohort of patients who underwent free flap reconstruction using conventional dissection techniques, which included electrocautery and LIGACLIP clips, was identified. These represented the most recent comparable cases, to minimize selection bias. To ensure comparability, patients in the conventional group were matched as closely as possible to the HARMONIC Shears group based on the free flap type and orthopedic procedure carried out during the same “fix and flap” operation. However, perfect matching was not always possible due to the variability inherent in trauma presentations.

Timing of reconstruction varied depending on patient optimization and orthoplastic coordination. Most reconstructions were performed within the 1st week post-injury, following completion of debridement and skeletal stabilization. All procedures in the HARMONIC Shears group were performed by a single consultant plastic surgeon with registrar assistance, whereas the conventional group included cases performed either by the same consultant as the HARMONIC Shears group or by another colleague with a subspecialty interest in lower limb reconstruction, both with registrar assistance.

### Study outcomes

The primary outcome was the difference in total operative time between patients undergoing free flap reconstruction using HARMONIC Shears vs conventional techniques. Secondary outcomes included flap survival and incidence of postoperative complications (infection, wound dehiscence, donor site morbidity, and reoperation). A cost analysis was also conducted to estimate the financial impact of differences in operative time, consumable use, and hospital stay costs between the two techniques.

### Inclusion and exclusion criteria

Eligible participants were adults (≥18 years) who underwent primary soft tissue free flap reconstruction for lower limb trauma within the study period, with no upper age limit applied. Exclusion criteria included previous reconstructive attempts on the affected limb and reconstruction for non-traumatic etiologies (e.g., oncologic or chronic wounds).

### Data collection

Data were collected from the electronic medical records system (EPIC), including operative notes and theater logs. Variables included total operative time (from incision to closure), flap type, details of concomitant orthopedic procedures, number of perforators dissected, ischemia time (from flap division to completion of vascular anastomosis), and postoperative outcomes (flap viability, complications, and length of stay).

### Cost analysis

A cost analysis was conducted to evaluate the economic impact of using the HARMONIC Shears. Theater time costs were calculated using the institution’s standard per-hour rate for operating room usage, while inpatient stay costs were based on the per-night rate for a general surgical ward. Consumable costs were obtained from hospital procurement records and compared between groups, including the HARMONIC FOCUS™+ Shears disposable handpiece and LIGACLIP™ ligature clips (Ethicon, Johnson & Johnson MedTech).

### Statistical analysis

All statistical analysis was performed using SPSS version 30 statistical software package (SPSS, Armonk, NY: IBM Corporation). Descriptive statistics were used to summarize patient demographics, operative characteristics, and outcomes. Continuous variables are presented as means ± standard deviations (SD), and categorical variables as counts. Comparisons between the HARMONIC Shears and conventional dissection groups were made using the unpaired *t*-test for continuous data and Fisher’s exact test for categorical variables. *p* < 0.05 was considered significant.

## Results

### Patient demographics

From January 01 2024 to June 30 2025, nine patients underwent free flap reconstruction for lower limb trauma using the HARMONIC Shears at our institution. A comparator cohort of nine patients who underwent free flap reconstruction using conventional dissection techniques was identified.

Patient demographics and injury characteristics are summarized in Table 1. Mean age was 36.4 years (SD = 16.0) in the HARMONIC Shears group and 44.1 years (SD = 16.0) in the conventional group (*p* = 0.32). All patients in the HARMONIC Shears group were male; the conventional group comprised seven males and two females. Body mass index (BMI) was comparable between groups (27.8 ± 5.9 vs 26.7 ± 2.9; *p* = 0.60). The median American Society of Anesthesiologists Physical Status Classification System (ASA) score in both groups was 2 (range 1–3). Seven of nine patients in the HARMONIC Shears group and six of nine patients in the conventional group were smokers. Comorbidities were present in one patient in the HARMONIC Shears group (hypertension, hyperlipidemia) and two in the conventional group (spinal stenosis, pacemaker for bradycardia)

**Table 1:** Patient demographics and injury characteristics.

Variable	HARMONIC FOCUS™+ Shears group (*n* = 9)	Conventional group (*n* = 9)	*p*-value
Age (years)	36.44 ± 16.03	44.11± 16.02	0.32
Sex (M/F)	9/0	7/2	
BMI	27.84 ± 5.86	26.66 ± 2.94	0.60
ASA Score	2 (1- 3)	2 (1–3)	
Smoker	7	6	
Comorbidities	1 (hypertension, hyperlipidemia)	2 (spinal stenosis, pacemaker)	
Mechanism of injury	Motorbike vs car: 4 Football tackle: 2 Car vs car: 1 Pedestrian vs car: 1 Fall from 1st storey window: 1	Motorbike vs car: 3 Car vs car: 2 Cyclist vs motorbike: 1 Fall from 1st storey window: 1 Slip on floor: 1 Fall down stairs: 1	
Lower limb fracture type	Distal tibia fibular fracture: 4 Tibia fibular diaphysis fracture: 4 No underlying fracture: 1	Distal tibia fibular fracture: 5 Tibia fibular diaphysis fracture: 3 Distal and proximal tibia fibular fracture: 1	

Patient demographics, comorbidities, injury mechanisms, and fracture types in the HARMONIC FOCUS™+ Shears group vs the conventional dissection group. Continuous variables are presented as mean ± standard deviation or median (range), and categorical variables are presented as counts. No statistically significant differences were observed between groups in baseline characteristics.

The most common mechanism of injury in both groups was motor vehicle-related trauma. Lower limb fractures predominantly involved the distal tibia-fibula or the diaphysis, with no significant differences in fracture patterns between groups.

### Operative characteristics

A range of free flap types was utilized in both groups, most commonly the ALT flap. Detailed operative characteristics are presented in Table 2. In cases without concomitant orthopedic procedures, operative time was significantly shorter in the HARMONIC Shears group compared to the conventional group (262.8 ± 46.3 min vs 364.2 ± 53.9 min, *p* = 0.012). When orthopedic procedures were performed during the same operation, including squaring off bone ends, syndesmotic screw placement, and intramedullary (IM) nailing, operative times remained comparable between groups (387.0 ± 36.5 min vs 394.7 ± 62.6 min, *p* = 0.86). Two additional complex reconstructions (MSAP (medial sural artery perforator) and saphenous artery flaps with IM nailing) were performed, with operative times of 423 and 419 min, respectively. When all cases were combined, the HARMONIC Shears group demonstrated a shorter mean total operative time compared to the conventional group (322.0 ± 81.1 min vs 380.4 ± 53.5 min), though this difference was not statistically significant (*p* = 0.09).

**Table 2:** Flap type, concomitant orthopedic procedures, and operative time in HARMONIC FOCUS™+ Shears and conventional dissection groups.

Variable	HARMONIC FOCUS™+ Shears Group	Conventional group	*p*-value
Free flap with no orthopedic procedure in same operation			
Flap type	ALT	ALT	
	ALT	ALT	
	ALT	ALT	
	ALT	ALT	
	TFL	ALT	
Operative time (min)	262.8 ± 46.3	364.2 ± 53.9	**0.012**
Free flap with orthopedic procedure in same operation			
Flap type and orthopedic procedure	ALT, squaring off bone ends	ALT, squaring off bone ends	
	ALT, syndesmotic screw	ALT, IM nail	
	TFL, IM nail	ALT, IM nail	
Operative time (min)	387 ± 36.5	394.7 ± 62.6	0.86
	MSAP, IM nail	Saphenous artery flap, IM nail	
Operative time (min)	423	419	
Total operative time (min)	322.0 ± 81.1	380.4 ± 53.5	0.09

Summary of free flap types, presence of orthopedic procedures performed during the same operation, and mean operative times in patients undergoing lower limb free flap reconstruction using the HARMONIC FOCUS™+ Shears vs conventional dissection techniques. Operative time is presented as mean ± standard deviation. Abbreviations: ALT, anterolateral thigh flap; TFL, tensor fascia lata flap; IM nail, intramedullary nail; MSAP, medial sural artery perforator flap.

Any *p*-value that is statistically significant we tend to bold to highlight its importance.

No significant difference was observed in the number of perforators dissected between the HARMONIC Shears and conventional dissection groups (mean 1.44 ± 0.52 vs 1.33 ± 0.50, respectively; *p* = 0.65). Similarly, ischemia times were comparable between groups, with a mean of 44.8 ± 11.6 min in the HARMONIC Shears group and 49.5 ± 12.5 min in the conventional group (*p* = 0.44).

### Flap survival and postoperative outcomes

Postoperative outcomes are summarized in Table 3. All nine patients in the HARMONIC Shears group achieved successful flap survival, compared with six of nine patients in the conventional group (*p* = 0.21). Of the three failed flaps, two were due to venous pedicle thrombosis and one due to combined arterial and venous pedicle thrombosis. Infection, wound dehiscence, and donor site morbidity occurred in up to three patients in the HARMONIC Shears group and in fewer patients in the conventional group; however, these differences were not statistically significant. Three patients in the conventional group required reoperation for debridement of nonviable flap tissue, followed by redo of the free flap or application of vacuum-assisted closure (VAC) dressing with split-thickness skin graft (SSG). Two patients in the HARMONIC Shears group underwent reoperation for debridement of a dehisced flap edge and subsequent SSG. The mean length of hospital stay was shorter in the HARMONIC Shears group (23.7 ± 13.7 days) compared to the conventional group (32.2 ± 20.0 days; *p* = 0.31), although this was also not statistically significant.

**Table 3:** Postoperative complications and length of hospital stay in HARMONIC FOCUS™+ Shears vs conventional dissection groups.

Post-op complications	HARMONIC FOCUS™+ Shears group (*n* = 9)	Conventional group (*n* = 9)	*p*-value
Flap survival	9	6	0.21
Infection	1	0	1.00
Wound dehiscence	3	0	0.21
Donor site morbidity	1 (infection)	2 (infection, seroma)	1.00
Return to theater	2 For washout and debridement of dehisced wound, SSG	3 For exploration and debridement of failed free flap, re-do of free flap	1.00
Length of stay (days)	23.7 ± 13.7	32.2 ± 20.0	0.31

Comparison of postoperative outcomes between patients who underwent free flap reconstruction using HARMONIC FOCUS™+ Shears vs conventional dissection techniques. Categorical data are presented as counts, and length of stay is reported as mean ± standard deviation. Abbreviations: SSG, split thickness skin graft.

### Cost analysis

Theater time was costed at £1746 per hour, based on institutional rates encompassing the fees for surgeons and anesthetists, as well as theater space and consumables. The HARMONIC FOCUS™+ Shears had a unit cost of £397.75. For cases performed using conventional dissection techniques, the LIGACLIP cost was £60.28 per case. The cost of an inpatient stay on a general surgical ward was calculated at £716 per night.

In free flap reconstructions without a concomitant orthopedic procedure, the average operative time was 101.4 min shorter in the HARMONIC Shears group, corresponding to a theater cost saving of £2950.74. After accounting for the cost of the HARMONIC Shears device and LIGACLIP consumables, this resulted in an average overall saving of £2613.27 per case in this subgroup. Across all free flap reconstructions in the study, the HARMONIC Shears group demonstrated a mean operative time reduction of 58.4 min, equating to £1699.44 in theater cost savings. After factoring in consumable costs, the average overall saving in this group was £1361.97 per case. The average hospital stay was 8.5 days shorter in the HARMONIC Shears group, translating to an additional inpatient cost saving of £6086.

When combining savings from both operative time and hospital stay, lower limb free flap reconstructions without a concomitant orthopedic procedure performed using the HARMONIC Shears achieved a total cost saving of £8699.27 per case. When including all lower limb free flaps, with or without a concomitant orthopedic procedure, the total saving was £7447.97 per case.

## Discussion

The HARMONIC FOCUS™+ Shears is an advanced ultrasonic energy device designed to deliver simultaneous tissue dissection, hemostasis, and vessel sealing with precision. Ultrasonic vibrations are converted into mechanical energy at the active blade, enabling tissue cutting, while concurrent frictional heat induces protein denaturation and vessel coagulation. The curved fine tip and slim profile of the HARMONIC FOCUS™+ Shears make it particularly suitable for use in confined anatomical spaces or near critical structures, which is especially useful during free flap harvest. The device can seal vessels up to 5 mm in diameter while maintaining excellent dissection accuracy.^1^^,^^4^ To illustrate its application, we have included a supplementary video demonstrating its use during flap elevation and perforator dissection (Video 1).

This study is, to our knowledge, the first to evaluate the HARMONIC FOCUS™+ Shears in free flap reconstruction for lower limb trauma. The findings demonstrated significant reductions in operative time where no concurrent orthopedic procedures were performed, with a trend toward shorter operative duration overall. Although not statistically significant, flap survival was higher in the HARMONIC Shears group, and the higher reoperation rate in the conventional dissection group reflected this difference. Postoperative complication rates, including infection, wound dehiscence, and donor site morbidity, were slightly higher in the HARMONIC Shears group, though again none of these differences were significant. Overall, these findings suggest that the use of the HARMONIC Shears may improve operative efficiency without evidence of a significant increase in complications compared with conventional techniques. However, given the small cohort, these results should be interpreted with caution, particularly regarding trends in operative time and flap outcomes.

The number of perforators dissected and flap ischemia times were comparable between groups, indicating that the use of the HARMONIC Shears does not compromise the technical quality of flap harvest or delay revascularization. The observed reduction in mean hospital stay with the HARMONIC Shears, though not statistically significant, may reflect possible benefits in recovery related to shorter exposure of general anesthetic during surgery, as well as improved resource utilization. The shorter stay may also relate to the higher flap success rate in this group, as patients with uncomplicated recoveries typically require less prolonged inpatient care.

These findings are concordant with broader surgical literature. The utility of ultrasonic energy devices is well established in several disciplines for reduced operative time, improved hemostasis, and diminished thermal injury compared to electrosurgical methods.^2^^,^^13^, ^14^, ^15^, ^16^ Published studies in head and neck and breast reconstruction support similar conclusions; Albert et al.^7^ described reduced flap harvest time, blood loss, and costs using ultrasonic dissection in forearm and fibula flaps, while Lee et al.^12^ reported reduced DIEP flap harvest time with HARMONIC Shears compared to bipolar cautery.

Notably, the literature addressing use for complex lower limb trauma remains limited. This study thus provides novel data supporting the HARMONIC Shears as a viable, potentially advantageous alternative to conventional approaches in this setting. While this study was conducted at a single tertiary center with a limited sample size, the principles demonstrated may be applicable to other reconstructive settings. The use of the HARMONIC Shears can potentially streamline flap harvest and improve operative efficiency in lower limb trauma reconstruction, particularly in high-volume microsurgical units. However, outcomes are likely to vary depending on case complexity and surgeon experience. Larger, preferably multi-center, studies are warranted to confirm these preliminary findings and expand generalizability.

In addition to operative efficiency, our study demonstrated a substantial cost saving associated with the use of the HARMONIC Shears, ranging from £7447.97 to £8699.27 per case. These savings reflect both reduced theater utilization, driven by shorter operative times, and shorter inpatient hospital stays. From an NHS perspective, this represents a meaningful reduction in resource burden for major reconstructive cases. Moreover, reduced operative time may also confer patient-centered benefits, including shorter exposure to general anesthesia and potentially faster postoperative recovery.

This study has several limitations. The small sample size limits statistical power, particularly in detecting differences in secondary outcomes such as flap survival and postoperative complications. Although patients were matched by flap type and the presence of a concomitant orthopedic procedure, perfect matching was not always possible due to the heterogeneity of trauma presentations. Importantly, total operative time was used as the primary outcome, which included time for orthopedic procedures in four patients in each group. The duration of these procedures was not recorded separately, and the HARMONIC Shears was not used for their execution, limiting the precision with which device efficacy can be assessed. Future studies should record flap harvest time separately. All HARMONIC Shears cases were performed by a single consultant surgeon, whereas the conventional group included cases performed by an additional surgeon. This introduces potential performance bias, which may have influenced operative times independently of the device itself. The HARMONIC FOCUS™+ Shears also has a learning curve, and outcomes in this study reflect use by experienced surgeons; wider adoption in routine practice may require additional training to achieve similar efficiency and safety.

Furthermore, the longer operative times and higher flap failure rate observed in the conventional dissection group may reflect the greater complexity of these cases rather than differences in dissection technique. The extended hospital stay and higher associated costs in this group may similarly be a consequence of more complex injuries, requiring additional postoperative care, monitoring, interventions, or rehabilitation before discharge. Finally, the cost analysis was based on institutional estimates and conservative assumptions about consumable use, which may limit generalizability.

## Conclusion

HARMONIC FOCUS™+ Shears appears to be a safe and feasible tool for lower limb free flap reconstruction following trauma. Preliminary data suggest potential benefits in operative efficiency and cost reduction without compromising flap survival or increasing complication rates. Future larger-scale, prospective studies are warranted to confirm these findings and further delineate the clinical and economic impact of ultrasonic dissection in complex lower limb reconstruction.

## Financial disclosure statement

No financial support, benefits, or incentives were received from any commercial entity related to this study. This research did not receive any external funding and incurred no additional costs beyond standard clinical care.

## Declaration of competing interest

The authors declare no conflicts of interest.

## Appendix Supplementary materials

Video 1: Intraoperative footage demonstrating branch ligation of an anterolateral thigh free flap pedicle using HARMONIC FOCUS™+ Shears. Image, video 1 dummy alt text
